# Ginsenoside Rg3 suppresses FUT4 expression through inhibiting NF-κB/p65 signaling pathway to promote melanoma cell death

**DOI:** 10.3892/ijo.2015.3057

**Published:** 2015-06-18

**Authors:** Xiu Shan, Li Li Tian, Yu Mei Zhang, Xiao Qi Wang, Qiu Yan, Ji Wei Liu

**Affiliations:** 1 Department of Oncology, The First Affiliated Hospital of Dalian Medical University, Dalian, Liaoning 116011, P.R. China; 2 Liaoning Provincial Core Lab of Glycobiology and Glycoengineering, Department of Biochemistry and Molecular Biology, Dalian Medical University, Dalian, Liaoning 116044, P.R. China; 3 Department of Dermatology, Northwestern University’s Feinberg School of Medicine, Chicago, IL 60611, USA

**Keywords:** Rg3, NF-κB, fucosyltransferase IV, apoptosis, melanoma

## Abstract

Abnormal glycosylation is catalyzed by the specific glycosyltransferases and correlates with tumor cell apoptosis. Increased fucosyltransferase IV (FUT4) is seen in many types of cancer, and manipulating FUT4 expression through specific signaling pathway inhibits cell growth and induces apoptosis. NF-κB is known playing a vital role to control cell growth and apoptosis. Ginsenoside Rg3 is an herbal medicine with strong antitumor activity through inhibiting tumor growth and promoting tumor cell death. However, whether Rg3-induced inhibition on tumor development involves reduced NF-κB signaling and FUT4 expression remains unknown. In the present study, we found that Rg3 suppressed FUT4 expression by abrogating the binding of NF-κB to FUT4 promoter through inhibiting the expression of signaling molecules of NF-κB pathway, reducing NF-κB DNA binding activity and NF-κB transcription activity. NF-κB inhibitor (Bay 11-7082) or knocking down p65 expression by p65 siRNA also led to a significant decreased FUT4 expression. In addition, Rg3 induced apoptosis by activating both extrinsic and intrinsic apoptotic pathways. Moreover, in a xenograft mouse model, Rg3 downregulated FUT4 and NF-κB/p65 expression and suppressed melanoma cell growth and induced apoptosis without any noticeable toxicity. In conclusion, Rg3 induces tumor cell apoptosis correlated with its inhibitory effect on NF-κB signaling pathway-mediated FUT4 expression. Results suggest Rg3 might be a novel therapy agent for melanoma treatment.

## Introduction

Malignant melanoma is the leading cause of skin cancer-associated mortality, the median survival time of patients with stage IV melanoma is less than 1 year and 5-year survival rate is less than 10% (1). Incidence and mortality rates of melanoma have increased steadily during the past four decades (2,3). Patients with early stages of melanoma are curable with surgery. However, once metastasis is established, it is difficult to treat and the mortality rate is high due to the lack of effective treatment. Despite significant improvement in immunotherapy and targeted therapy in recent years, only a section of patients with metastatic melanoma benefit for a short of period of response because of drug resistance and high toxicity (4). Hence, identification of potential new target drugs and development of effective therapeutic strategies are the utmost importance to reduce melanoma-related mortality.

NF-κB represents a family of inducible transcription factors involved in the maintenance of various cellular functions, such as cell cycle progression, apoptosis, inflammatory and immune responses (5). In cancer cells, NF-κB is proposed to play an important role in tumorigenesis, promoting cell proliferation, migration, angiogenesis and anti-apoptotic effects. The regulation of NF-κB activation is determined by its subcellular localization. In unstimulated cells, NF-κB formed a complex in the cytoplasm with its inhibitor IκB. Cell stimulation accompanied by IκB phosphorylated proteins and ubiquitinated, thus liberating NF-κB from IκB complexes (6). The active NF-κB translocates into the nucleus and activates the expression of specific κB enhancer gene (5). Aberrant activation of NF-κB has been reported in various types of cancer, including pancreatic, prostate and colon cancer (7,8). NF-κB is found constitutively activated in human melanoma cells, and upregulation of the NF-κB levels promotes the progression of melanoma and increases metastatic potential (9,10). Inhibition of NF-κB activation in human melanoma cells enhanced radio-sensitivity, induced apoptosis and inhibited invasion (11,12).

Protein glycosylation plays an important role in pathophysiological steps of tumor progression, including tumor proliferation, invasion, metastasis and angiogenesis (13). Fucosylation is one of the important steps in protein glycosylation. Fucosyltransferases (FUTs) are the key enzymes catalyzing the synthesis of fucosylated glycans. At least eight of 1, 3/4-FUT genes have been identified, they are FUT3, 4, 5, 6, 7, 9, 10 and 11 (14,15). Among these FUTs, FUT4 catalyzes the synthesis of α1, 3-fucosylation of Lewis Y. Several reports have shown that FUT4 is overexpressed in many types of cancer, including colorectal, gastric and lung cancer (16–18). Furthermore, increased FUT4 expression has been implicated to promote cell proliferation, metastasis and anti-apoptosis (19–21). However, whether FUT4 is increased in melanomas, and whether FUT4 and NF-κB signaling pathway is involved in 20 (R)-Ginsenoside Rg3-induced melanoma cell apoptosis are largely unknown.

Ginseng, is a well-known herbal medicine, used for thousands of years alone or in combination with other herbal ingredients, such as invigorant, cardiotonic, and drugs for anti-inflammatory, antitumor and immune stimulation. Rg3 is a monomer extracted from ginseng and has a broad spectrum of antitumor activities. Rg3 inhibits cell proliferation and induces apoptosis in colon and gastric cancer (22,23). In addition, Rg3 enhances the chemosensitivity of patients to docetaxel and cisplatin with prostate and colon cancer (24,25). Moreover, Rg3 has been shown to inhibit tumor angiogenesis and induces cancer cell apoptosis in liver carcinomas (26). However, the antitumor mechanism of Rg3, FUT4 and NF-κB pathway on human melanoma remains unclear.

In the present study, we demonstrated, both *in vitro* and *in vivo*, that Rg3 suppressed FUT4 expression by inhibiting NF-κB signaling pathway, by which it induced melanoma apoptosis. In conclusion, our results suggest that Rg3 deactivates NF-κB signaling pathway to downregulate FUT4 playing an important role in inhibiting melanoma progression.

## Materials and methods

### Ethics statement

All animal work performed in the present study was approved by the Animal Ethics Committee of the Dalian Medical University. The detail protocols and experimental processes conformed to the Experimental Animal Management Regulations of Dalian Medical University.

### Antibodies and reagents

Dulbecco’s modified Eagle’s medium (DMEM), fetal bovine serum (FBS), TRIzol and Lipofectamine™ 2000 reagents were purchased from Invitrogen (Camarillo, CA, USA). Rg3 was provided by Dalian Fusheng Pharmaceutical Co., Ltd. (Dalian, China). It was diluted with cell culture media to final concentration. Cell Counting kit-8 (CCK-8) was purchased from Dojindo Laboratories (Kumamoto, Japan). Annexin V/FITC kit was purchased from Nanjing KeyGen Biotech Co., Ltd. (Nanjing, Jiangsu, China). Antibodies specific for PARP, caspase-3, -8, -9, Bcl-2, survivin, Bax, FUT4, β-actin, HRP-conjugated anti-rabbit and anti-mouse antibodies were purchased from Proteintech Group (Wuhan, China). Antibodies specific for p-p65, p65, pIκBα, IκBα, pIKKα/β and IKKα/β were purchased from Cell Signaling Technology (Boston, MA, USA). p65 siRNA was purchased from the Shanghai GenePharma Co. (Shanghai, China). NF-κB inhibitor (Bay 11-7082) was purchased from Selleck Chemicals (Houston, TX, USA). Nuclear extract kit and EMSA kit were purchased from Thermo Fisher Scientific (Waltham, MA, USA). ChIP kit was purchased from Millipore (Billerica, MA, USA). The enhanced chemiluminescence (ECL) assay kit was purchased from Amersham Biosciences (Pittsburgh, PA, USA).

### Cell culture

Human melanoma cell lines A375P, A375M, C8161, Mevo and SK-MEL-28 were gifted from Dr Mary Hendrix (Stanley Manne Children’s Research Institute Northwestern University’s Feinberg School of Medicine, Chicago, IL, USA) and were grown in DMEM with 10% FBS, 100 U/ml penicillin and 100 μg/ml streptomycin; maintained at 37°C under 5% CO_2_ in humidified air.

### Cell viability assay

Melanoma cells were plated at a density of 2,000 cells/well in 96-well plates, and treated with various concentrations of Rg3 for 24 h the following day. Cell viability was evaluated in cells using CCK-8 kit according to the manufacturer’s instructions. Briefly, 10 μl of the CCK-8 solution was added to cell cultures for the designated times. Plates were incubated for 1 h with CCK-8 solution at 37°C. The optical density (OD) was read at 450 nm absorbance on a microplate reader (Bio-Rad Laboratories, Hercules, CA, USA).

### Colony forming assay

Cells (1×10^3^ cells/well) were plated in 6-well plates containing DMEM with 10% FBS at 37°C. After 24 h, cells were treated with different concentrations of Rg3 (0, 25, 50, 75 and 100 μg/ml) for 24 h and then cells were allowed to grow for 10 days in the absence of Rg3. Cells were fixed and stained with crystal violet (0.5%) for 20 min at room temperature. Images were captured with the inverted microscope (Olympus IX71; Olympus, Tokyo, Japan).

### Apoptosis assay

Apoptosis was assessed by Annexin V-binding analysis of flow cytometry. For flow cytometric analysis, cells (1×10^5^) were seeded in 6-cm dishes overnight before treated with various concentrations of Rg3 for 24 h. Both adherent and floating cells were harvested and combined, washed twice with PBS, resuspended in 500 μl of binding buffer, and stained using an Annexin V-FITC/PI kit according to the manufacturer’s instructions. After incubation in the dark for 30 min, the cells were analyzed using FACScalibur instrument (FACSCalibur; BD Biosciences, San Jose, CA, USA). All experiments were performed in duplicate and reproducibility was checked in three independent experiments.

### Quantitative real-time PCR analysis

Total RNA was extracted using TRIzol (Invitrogen) according to the manufacturer’s protocol. RNA was reverse transcribed into cDNA using PrimeScript™ RT reagent kit (Takara, Tokyo, Japan). The FUT4 primers were 5′-AAGGTCCAGGCCCACTGAAG-3′ (forward) and 5′-CAGTTCAGGTGACAGAGGCTCAA-3′ (reverse); the GAPDH primers were 5′-ATGGGGAAGGTGAAGGTCG-3′ (forward) and 5′-GGGGTCATTGATGGCAACAATA-3′ (reverse). Real-time quantitative PCR reactions were performed with Applied Biosystems StepOne Real-time PCR system (Life Technologies, Carlsbad, CA, USA). Relative FUT4 mRNA levels were normalized with GAPDH and calculated using 2^−ΔΔCT^ method.

### Western blot analysis

Cells were washed with PBS (pH 7.4), and incubated with 2× concentrated electrophoresis sample buffer (125 mM Tris-HCl, pH 6.8, 5% glycerol, 2% SDS, 1% β-mercaptoethanol) for 30 min on ice. Protein concentration was determined with Coomassie protein assay reagent using bovine serum albumin as a standard. Total protein (50–70 μg) from the whole cell lysates was separated by 12% SDS-PAGE and proteins separated in the gel were transferred electrophoretically onto nitrocellulose membrane (Millipore, Billerica, MA, USA), incubated with TTBS (50 mM Tris HCl, pH 7.5, 0.15 M NaCl, 0.1% Tween-20) containing 5% fat-free dry milk for 2 h followed by overnight incubation with the appropriate primary antibodies at the dilutions recommended by the suppliers at 4°C. After incubation with an HRP-conjugated appropriate secondary antibody, ECL (enhanced chemiluminescence) detection system (Bio-Rad Laboratories) was used to visualize immunoreactive bands.

### Electrophoretic mobility shift assay (EMSA)

Cells were treated with Rg3 (75 μg/ml) for 24 h. The DNA binding activities of NF-κB in nuclear extracts were assessed using the EMSA kit with biotin-labeled double-stranded NF-κB oligonucleotides (Beyotime, Nantong, China). The sequences of the oligonucleotides adopted were as follows: 5′-CGCTTGATGACTCAGCCGGAA-3′ and 3′-GCGAACTACTGAGTCGG CCTT-5′. Briefly, nuclear extracts (8 μg/sample) were incubated with the oligonucleotides in reaction buffer for 20 min. Protein DNA complexes were separated on a 6.5% non-denaturing acrylamide gel, transferred to positively charged nylon membranes, and immobilized by UV cross-linking for 10 min. Band shifts were detected by chemiluminescence method with a detection system (Bio-Rad Laboratories).

### Luciferase reporter assay

The NF-κB luciferase activity was determined in A375P and C8161 cells after co-transfected, using Lipofectamine 2000 reagent, with 2 μg of NF-κB luciferase plasmids and 0.2 μg of pGL3, which constitutively expressed *Renilla* luciferase. Twenty-four hours after transfection, the cells were treated with Rg3 (50 or 75 μg/ml as indicated) for 24 h. The luciferase activity was assayed using the Dual Luciferase reporter assay system Berthold Technologies (Bad Wildbad, Germany). Firefly luciferase activity was measured and the reading was normalized to *Renilla* luciferase activity, which served as an internal control for transfection efficiency.

### Chromatin immunoprecipitation

The chromatin immunoprecipitation (ChIP) assay was performed according to the manufacturer’s instruction using cells at ~80% confluent post Rg3 treatment for 24 h. In brief, 1% paraformaldehyde was added to the cell-culture medium and incubated at room temperature for 10 min to cross-link. The cells were then washed twice in cold phosphate-buffered saline, scraped and lysed in SDS lysis buffer containing protease inhibitor cocktail for 10 min at 4°C. The lysates were sonicated five times for 15 sec each time, and the debris was removed by centrifugation. A total of 10 μl of the supernatant was used as an input sample, and the remaining of the lysate were diluted 10-fold with a dilution buffer containing protease inhibitor cocktail followed by incubation with antibodies against specific transactivators or a non-immune rabbit IgG control overnight at 4°C. Immunoprecipitated complexes were collected using protein A/G plus agarose beads. The precipitates were extensively washed and incubated in an elution buffer at room temperature for 20 min. Cross-linking of protein-DNA complexes were reversed at 65°C for 5 h, followed by treatment with 100 mg/ml Proteinase K for 2 h at 45°C. DNA was extracted three times with phenol/chloroform and precipitated with ethanol. The pellets were resuspended in TE buffer and subjected to PCR amplification using specific FUT4 promoter primers (forward primer, CCATTCCCAGCACTGTCTATTTC and reverse primer, CCTACGGGTTGAATTTGAATTTCT. The resulting product of FUT4 was separated by 1.0% agarose gel electrophoresis.

### Xenograft tumor mouse models

Male nude mice (Balb/c-nu/nu, 4–6 weeks old) were obtained from the Animal Center (Dalian Medical University) and maintained under sterile conditions during the entire experimental period. Mice were divided into two groups (n=8/group) based on the treatment. A375P cells (2×10^6^) suspended in 0.2 ml PBS were injected subcutaneously into the right flank. After 7 days of tumor inoculation, mice were treated with Rg3 (20 mg/kg of body weight) or its vehicle control subcutaneously for 3 weeks with time interval of 48 h. Tumor volume was measured by Vernier calipers every other day after tumor was palpable. The tumor volume was calculated according to the formula (volume=1/2 length × width^2^). At the end of the experiment (at day 30), the tumor was removed and weighed.

### Statistical analysis

Each experiment was repeated 3 times and results are presented as the mean ± SEM. Unpaired Student’s t-tests were used to analyze differences between groups. P<0.05 was considered to be significant. The statistical software SPSS ver. 17 was used for analyzing the data.

## Results

### Rg3 inhibits human melanoma cell proliferation

**Figure 1:**
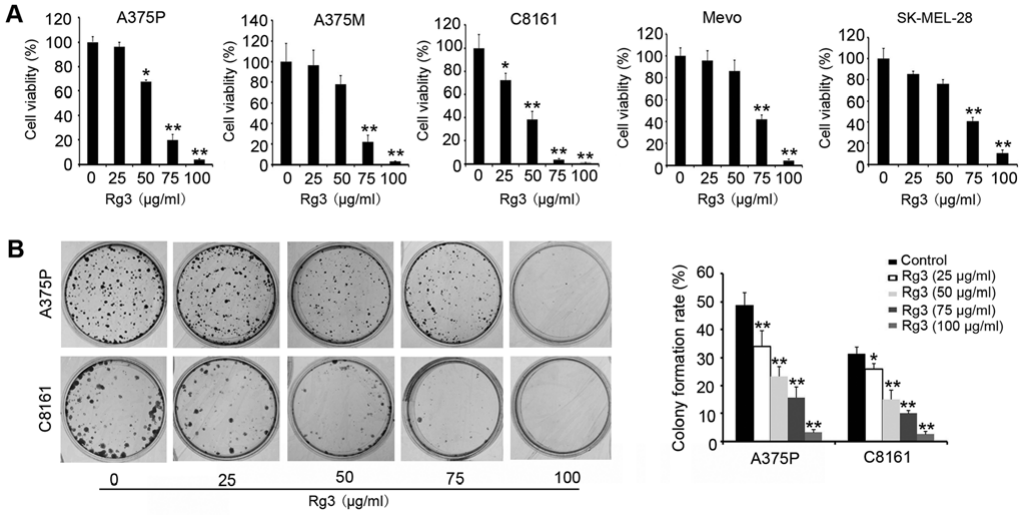
Rg3 inhibits human melanoma cell proliferation. (A) A375P, A375M, C8161, Mevo and SK-MEL-28 cells were treated with Rg3 (0, 25, 50, 75 and 100 μg/ml) for 24 h. Cell viability was determined by CCK-8 assay as described in Materials and methods. (B) Long-term colony formation assay of A375P and C8161 cells. Cells were grown in the absence or presence of Rg3 at the indicated concentrations (0, 25, 50, 75 and 100 μg/ml) for 10 days. Cells were fixed and stained with crystal violet. All results are representative of mean values from three separate experiments in triplicate. ^*^P<0.05; ^**^P<0.01.

To evaluate the effect of Rg3 on melanoma cell proliferation, we first treated various melanoma cells with different concentrations of Rg3 (0, 25, 50, 75 and 100 μg/ml) for 24 h. CCK-8 proliferation assay showed that Rg3 treatment significantly inhibited cell proliferation/viability in all cell lines tested (Fig. 1A). The IC_50_ values are in the range of 57–83 μg/ml. The sensitivity of the cell responses to Rg3 was C8161>A375P>A375M>SKMEL-28>Mevo. We selected the most sensitive cell lines, A375P and C8161, for further study. Colony forming assay revealed that Rg3 treatment led to a significant decrease in the number of colonies (P<0.05) (Fig. 1B). The effect of Rg3 on cell growth was dose-dependent. These results indicate that Rg3 has a cytotoxic effect on melanoma cells and inhibits cell growth.

### Rg3 induces human melanoma cell apoptosis

**Figure 2:**
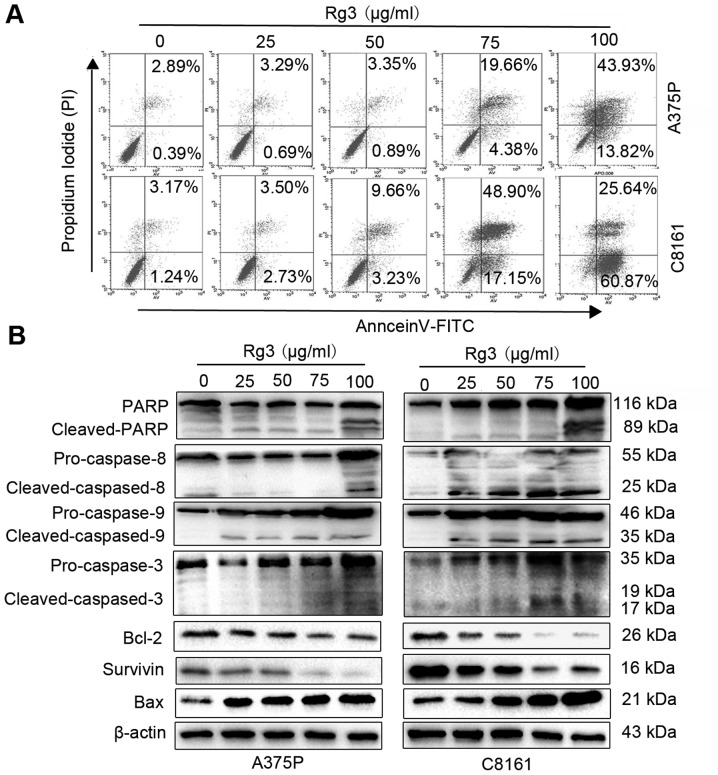
Rg3 induces human melanoma cell apoptosis. (A) A375P and C8161 cells were double stained with Annexin V/PI and analyzed by FACS analysis after 24-h treatment with Rg3 (0, 25, 50, 75 and 100 μg/ml). The percentage of Annexin V^+^/PI^−^ (early apoptotic cells, lower right), Annexin V^+^/PI^+^ (late apoptotic cells, upper right), Annexin V^−^/PI^−^ (viable cells, lower left) and Annexin V^−^/PI^+^ (necrotic cells, upper left), cells is shown. (B) A375P and C8161 cells were treated with Rg3 (0, 25, 50, 75 and 100 μg/ml) for 24 h, the expression levels of PARP, caspase-8, 9, 3, Bcl-2, survivin and Bax were analyzed by western blot analysis.

We investigated whether the observed Rg3 antitumor effect in human melanoma cells correlates with its effect on cell apoptosis. Human melanoma cell lines A375P and C8161 were treated with different concentrations of Rg3 for 24 h, followed by flow cytometric analysis after staining with Annexin V-FITC/PI. The results showed that treatment with Rg3 (0, 25, 50, 75 and 100 μg/ml) for 24 h led to concentration-dependent increases in apoptotic cells in both A375P and C8161 cells, from 3.28 and 4.41% in untreated control groups to 3.98 and 6.23% (25 μg/ml), 4.24 and 12.89% (50 μg/ml), 24.04 and 66.05% (75 μg/ml), and 57.75 and 86.51% (100 μg/ml), respectively (Fig. 2A). We further examined the expression of apoptotic marker proteins. As shown in Fig. 2B, Rg3 treatment effectively activated the expression of pro-apoptotic proteins (caspase-3, -8, -9 and Bax), decreased the expression of anti-apoptosis proteins (Bcl-2 and survivin) in a dose-dependent manner (Fig. 2B). In addition, Rg3 also activated PARP, a marker of apoptosis (Fig. 2B, top row). These results indicate that Rg3 induces human melanoma cells apoptosis.

### Rg3 inhibits the activation of the NF-κB signaling pathway

**Figure 3:**
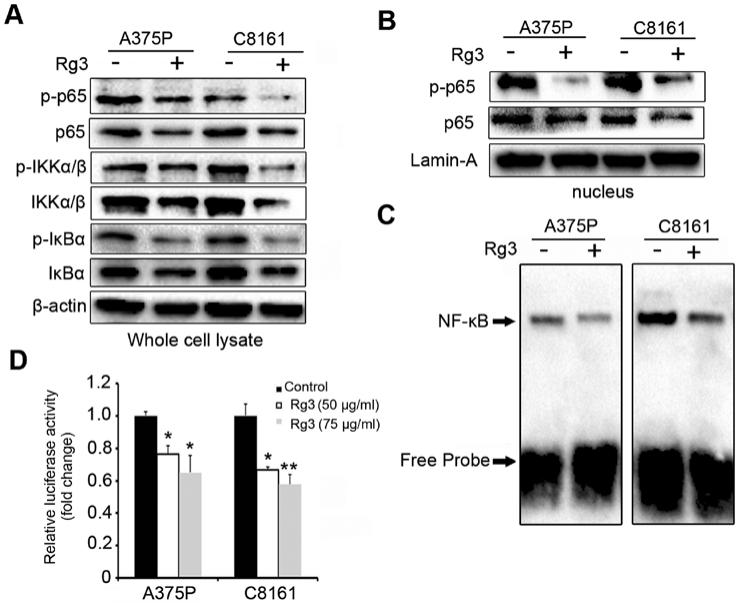
Rg3 inhibits the activation of NF-κB signaling pathway. (A) Analysis of phosphorylation and non-phosphorylation levels of NF-κB/p65, IKKα/β and IκBα levels by western blot analysis in A375P and C8161 cells after cells were treated with Rg3 (75 μg/ml) for 24 h. (B) Cells were treated with 75 μg/ml of Rg3 for 24 h. The nuclear levels of the proteins were detected by western blot assay. Lamin-A was used as nuclear loading control. (C) A375P and C8161 cells were preincubated with Rg3 (75 μg/ml) for 24 h. EMSA was performed in nuclear extract for DNA binding of activated NF-κB assay. (D) Cells were transfected with NF-κB luciferase reporter for 24 h, and then preincubated with Rg3 (75 μg/ml) for 24 h. Luciferase activity was measured in the cell lysate. Data are expressed as means ± SD. ^*^P<0.05; ^**^P<0.01.

Previous studies have demonstrated that NF-κB plays a critical role in apoptosis and cell survival, it is possible that NF-κB pathway might also be involved in Rg3-induced apoptosis in human melanoma cells. As shown in Fig. 3A, treatment with Rg3 (75 μg/ml) for 24 h led to a significant decrease in the phosphorylation of NF-κB/p65, IκBα and IKKα/β in both A375P and C8161 cells. Similarly, we also observed a decrease in the basal level of NF-κB/p65, IκBα and IKKα/β after Rg3 treatment (Fig. 3A). Moreover, Rg3 decreased the nuclear level of NF-κB/p65 and its phosphorylation (Fig. 3B), suggesting Rg3 also inhibited the nuclear translocation of NF-κB/p65. Subsequently, we investigated the effect of Rg3 on DNA-binding activity of NF-κB/p65. By EMSA assays, we found dramatically reduced NF-κB DNA binding in Rg3 (75 μg/ml) treated cells (Fig. 3C). To further investigate the effects of Rg3 on NF-κB activity, NF-κB-Luc was transfected into A375P and C8161 cells and the luciferase activity was measured. As shown in Fig. 3D, preincubation with Rg3 (50 and 75 μg/ml) significantly suppressed the NF-κB transcription activity (P<0.05 or <0.01). These results demonstrate the inhibitory effects of Rg3 on the NF-κB signaling pathway and the NF-κB DNA binding/transcription activities.

### Rg3 decreases FUT4 expression and inhibits NF-κB binging to FUT4 promoter

**Figure 4:**
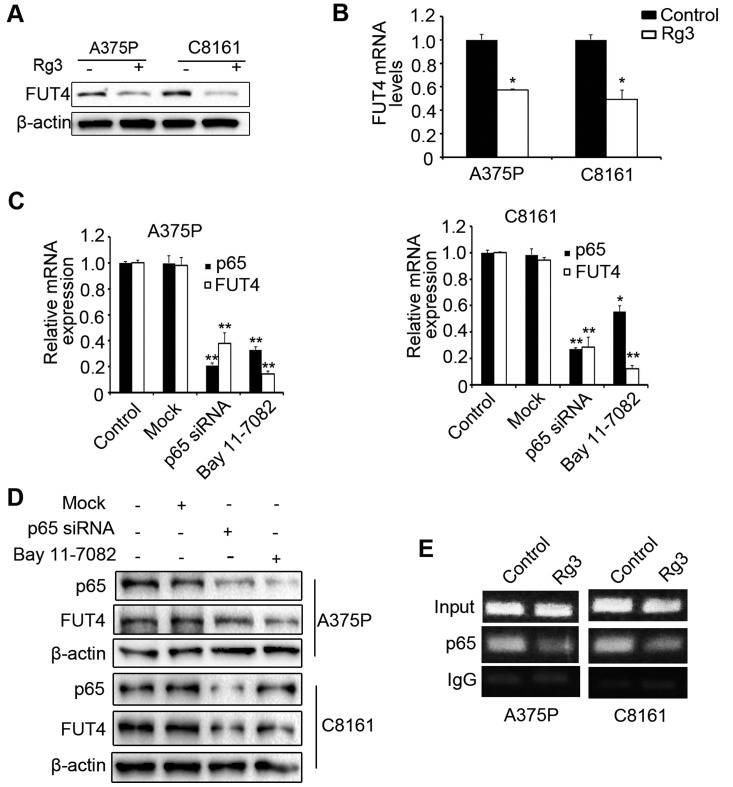
Rg3 decreases FUT4 expression and inhibits NF-κB binging to FUT4 promoter. FUT4 was detected by (A) western blotting and (B) real-time PCR in A375P and C8161 cells after treatment with Rg3 (75 μg/ml) for 24 h. p65 and FUT4 expression by (C) real-time PCR and (D) western blotting in A375P and C8161 cells. Control, untransfected cells; Mock, cells transfected with vector; p65 siRNA, cells transfected with p65 siRNA; cells were exposed to Bay 11-7082 (10 μM) for 24 h. (E) After treatment with Rg3 (75 μg/ml) for 24 h, chromatin in the treated cells was immunoprecipitated with antibodies to p65 and the FUT4 promoter region in the precipitated chromatin was amplified by PCR. Data are presented as the mean ± SD of three independent experiments. ^*^P<0.05; ^**^P<0.01.

A previous study showed that FUT4 was an anti-apoptosis protein. Increased expression of FUT4 inhibited cyclophosphamide-induced apoptosis in A431 cells (21). To examine whether the effect of Rg3-induced apoptosis was related to decreased expression of FUT4. A375P and C8161 cells were treated with Rg3 (75 μg/ml) for 24 h and FUT4 expression was examined. Results showed that treatment with Rg3 suppressed FUT4 expression in both protein (Fig. 4A) and mRNA (Fig. 4B) levels. These results suggest that Rg3 downregulates the expression of FUT4. To examine whether FUT4 expression is regulated by NF-κB signaling pathway, NF-κB signaling was disrupted by either knocking down p65 or treatment with NF-κB inhibitor, Bay 11-7082 (10 μM). Results showed that disrupting NF-κB downregulated the expression of FUT4 at both mRNA (Fig. 4C) and protein (Fig. 4D) levels. We further evaluated the effect of Rg3 on the binding activities of NF-κB to FUT4 promoter by ChIP assay. Results showed that treatment of cells with Rg3 for 24 h markedly inhibited the binding of NF-κB p65 subunits to the FUT4 chromatin structure as compared with the control (Fig. 4E). These results suggest that the inhibition of FUT4 expression by Rg3 was modulated by the NF-κB/p65 signaling pathway and FUT4 is a possible downstream target of NF-κB.

### Rg3 induces apoptosis by inhibiting both the NF-κB signaling and FUT4 expression

**Figure 5:**
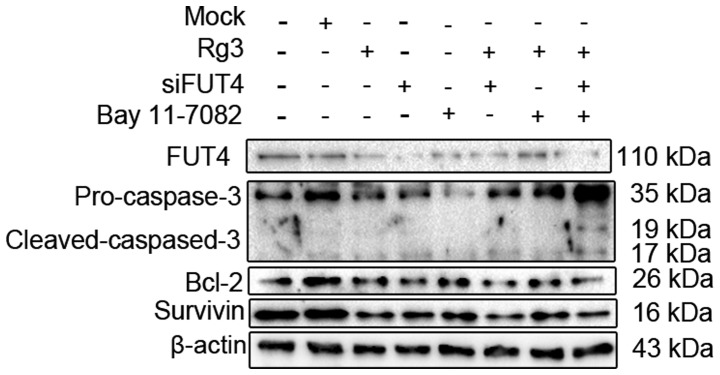
Rg3 induced apoptosis by inhibiting NF-κB signaling pathway with FUT4 downregulation. A375P cells were pretreated by FUT4 siRNA, Rg3 (75 μg/ml) or NF-κB inhibitor Bay 11-7082 (10 μM). Control, untransfected cells; Mock, cells transfected with vector. Western blot analysis of expression levels of FUT4, caspase-3, Bcl-2 and survivin.

To determine the role of NF-κB signaling pathway and the FUT4 expression in Rg3-induced apoptosis, A375P cells were incubated with Rg3 (75 μg/ml), FUT4 siRNA and Bay 11-7082 (10 μM) for 24 h, or preincubated with FUT4 siRNA 48 h, Bay 11-7082 (10 μM) 24 h, followed by Rg3 (75 μg/ml) treatment for 24 h. As shown in Fig. 5, treatment with Rg3, FUT4 siRNA and NF-κB inhibitor resulted in the increased expression of cleavage of caspase-3 by 2.0-, 2.5-and 1.2-fold, and decreased the expression of anti-apoptosis proteins Bcl-2 and survivin. While Rg3 combined with FUT4 siRNA and Bay 11-7082 showed higher effect on the expression of apoptosis-related proteins than either of them alone. This result suggested that Rg3-induced melanoma cell apoptosis was augmented by FUT4 siRNA and disrupted the NF-κB signaling pathway with FUT4 downregulation. Moreover, NF-κB signaling pathway blockage and FUT4 downregulation could be an effective approach in sensitization of the antitumor efficacy of Rg3 in human melanoma cells.

### Rg3 suppresses the growth of human melanoma xenografts

**Figure 6:**
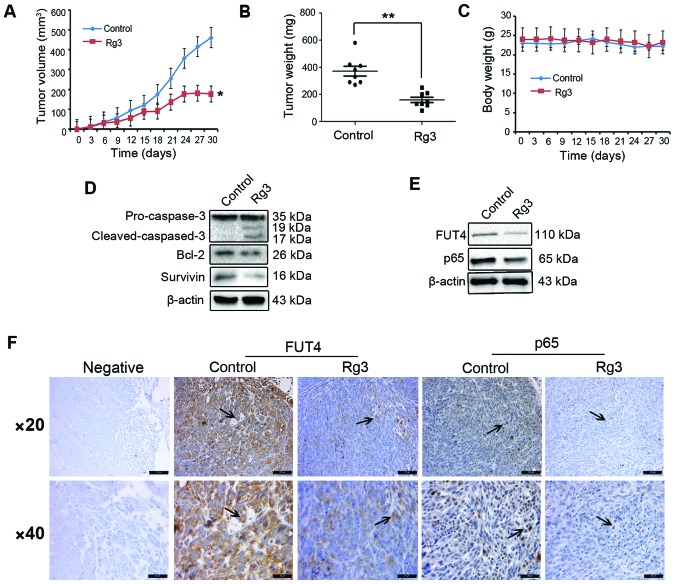
Rg3 suppresses the growth of human melanoma cancer xenografts. A375P cells xenografted nude mice were injected with vehicle (control) and Rg3 (20 mg/kg). The animal experiment was carried out for 30 days. (A) Tumor volumes after treatment with Rg3 in the xenografts (n=8 per group). (B) Tumor weight. (C) Body weight. (D and E) Western blot analysis of expression levels of caspase-3, Bcl-2, survivin and p65, FUT4 in lysates from the tumors in differently treated mice. (F) Immunohistochemical staining of FUT4 and p65 expression in xenograft tumor tissues. Arrows indicate the expression of FUT4 and p65 in different groups. Magnifications, ×200 and ×400. ^*^P<0.05; ^**^P<0.01.

The anticancer activity of Rg3 was further evaluated in A375P melanoma cell xenograft mouse model. As showed in Fig. 6A, treatment with Rg3 led to a significant inhibition of tumor growth by 53.56% (P<0.05). Tumor weight in groups treated with Rg3 (20 mg/kg) decreased by 52.86% compared with the control group (Fig. 6B, P<0.01). All animals survived during the experimental period, treatment with Rg3 (20 mg/kg) was well tolerated and there was no significant loss of body weight or any observable toxic effect in mice treated with Rg3 in comparison with the control (Fig. 6C). The regulation of apoptosis-related proteins in the xenograft tumor specimens was also assessed. As shown in Fig. 6D, Rg3 treatment led to a significantly increased expression of cleavage of caspase-3, and decreased expression of the anti-apoptosis proteins Bcl-2 and survivin in comparison to controls. Moreover, by western blot analysis and immunohistochemical staining, we found that the expression levels of FUT4 and p65 were significantly decreased with Rg3 treatment as compared with the control group (Fig. 6E and F).

## Discussion

It has been well established that Rg3 has strong antitumor activity. The abnormal activation of signal pathways is closely related to tumor cell growth, apoptosis and metastasis. Our previous study showed that Rg3-induced EGFR/MAPK pathway deactivation inhibiting melanoma cells proliferation via decreasing FUT4/LeY expression (27). It has been reported that Rg3 promotes apoptosis in human ovarian cancer through the PI3K/Akt pathways (28). Rg3 also sensitizes prostate cancer cells to docetaxel and other chemotherapeutics by inhibiting cell growth and inducing apoptosis via inhibition of NF-κB signaling pathway (24). Rg3 induced apoptosis in MDA-MB-231 cells by blocking NF-κB signaling via inactivation of ERK and Akt as well as destabilization of mutant p53 (29). In the present study, we demonstrated that Rg3 effectively inhibited melanoma cell growth and induced apoptosis in a dose-dependent manner, with IC_50_ range from 57 to 83 μg/ml. We discovered that Rg3 inhibited melanoma growth and induced apoptosis through inhibiting NF-κB/FUT4 signaling pathway and activating the extrinsic and the intrinsic apoptotic pathways both *in vitro* and *in vivo*.

FUT4, as a key enzyme involved in LeY synthesis, plays multiple roles in the regulation of various pathophysiologic processes. Emerging evidence has shown that FUT4 is a potential target to inhibit cell proliferation, survival, invasion and metastasis (19,20,29). Suppressing FUT1/4 expression blocked EGF-induced tyrosine phosphorylation of EGFR and MAPK and inhibited cancer growth (30); Yang *et al* (21) reported that FUT4 overexpression inhibited cyclophosphamide-induced apoptosis through activating the ERK/MAPK and PI3K/Akt signaling pathways. Moreover, FUT4 stimulates epithelial-mesenchymal transition (EMT) to promote tumor metastasis (20). FUT4 expression can be regulated in different levels, including modulation of FUT4 promoter activity, interfering gene transcription and/or translation. For example, in breast cancer, FUT4 transcription is regulated by HSF1 and Sp1 to inhibit cell proliferation (31); in HaCaT cells, FUT4 level is lower due to the higher methylation of CpG island in FUT4 promoter (32). Knocking down FUT1/4 expression by short interfering RNA technique dramatically reduced the expression of FUT1/4 and the end product of FUT4, LeY, by which to inhibit cell proliferation (30). In the present study, we found that NF-κB/p65 dependent transcriptional regulation of FUT4 inhibited cell proliferation and induced apoptosis in human melanoma cells. Suppression of p65 by either siRNA or the potent NF-κB inhibitor Bay 11-7082 inhibited FUT4 expression. To the best of our knowledge, this is the first report that NF-κB/p65 signaling regulates the expression of FUT4. These results suggest that FUT4 is one of the target molecules regulated by NF-κB/p65.

The transcription factor, nuclear factor-κB (NF-κB) plays significant roles in the regulation of cell growth, survival and apoptosis. NF-κB activation allows cancer cells to escape apoptosis, and attenuates the effect of chemotherapeutic agent-induced apoptosis (11,33). Previous studies have shown that abnormal activation of NF-κB correlates with numerous human malignancies, such as pancreatic, breast, colon cancer, T-cell leukemia and melanoma (34–37). NF-κB stimulates the expression of anti-apoptotic proteins, such as the inhibitor of apoptosis proteins and Bcl-2 family members, thereby promoting cell survival (38). Suppression of NF-κB activity significantly reduced cell growth, induced apoptosis and increased sensitivity to radiation-induced cytotoxicity (11). In the present study, we found that Rg3 not only inhibited the expression of NF-κB/p65, IκBα and IκB kinase (IKKα/β) activation, but also inhibited NF-κB DNA binding and transcription activity in human melanoma cells. Aberrant expression of FUT4 is known to correlate with the activation of NF-κB signaling pathway. Yang *et al* (20) showed that FUT4 knockdown led to decreased nuclear expression of NF-κB in breast cell lines. Cheng *et al* (39) found that, in MDR hepatocellular carcinoma cells, interference or overexpression of FUT4 decreased or increased the expression of NF-κB and NF-κB DNA-binding, respectively. Therefore, we hypothesized that NF-κB could also regulate FUT4 expression. In the present study, our data showed that Rg3 inhibited NF-κB translocation from cytosol to nuclear and the silencing of NF-κB/p65 not only significantly downregulated p65 expression, but also suppressed FUT4 expression. Moreover, Rg3 inhibited NF-κB/p65 binding to FUT4 promoter to abrogate FUT4 transcriptional activation, by which to reduce FUT4 expression promoting apoptosis.

Collectively, the present study demonstrates that Rg3 inhibits the growth of human melanoma cells and induces cell apoptosis both *in vitro* and *in vivo*. Moreover, downregulation of FUT4 expression by Rg3 induces cell apoptosis through inhibiting NF-κB signaling pathway. These results suggest that suppression of NF-κB/FUT4 activation by Rg3 may be a useful strategy in the prevention or treatment of melanoma. These findings provide strong evidence that Rg3 is a potential novel therapeutic for treatment of melanoma.

## Abbreviations

FUT4: fucosyltransferase IV
NF-κB: nuclear factor-κB
LeY: Lewis Y
EMT: epithelial-mesenchymal transition
siRNA: small interfering RNA
PARP: poly ADP-ribose polymerase
DAPI: 4,6-diamino-2-phenyl indole
EMSA: electrophoretic mobility shift assay
ChIP: chromatin immunoprecipitation
